# Supplementary material for: Effects of potent neutralizing antibodies from convalescent plasma in patients hospitalized for severe SARS-CoV-2 infection
Source: Nat Commun. 2021 May 27;12:3189. doi: 10.1038/s41467-021-23469-2 (PMC8160346; doi:10.1038/s41467-021-23469-2)
Supplement: Supplementary file 1 — Supplementary Information [file 41467_2021_23469_MOESM1_ESM.pdf]

Effects of Potent Neutralizing Antibodies from Convalescent Plasma in Patients Hospitalized for Severe SARS-CoV-2 Infection.

Supplementary Information

Supplementary Figures

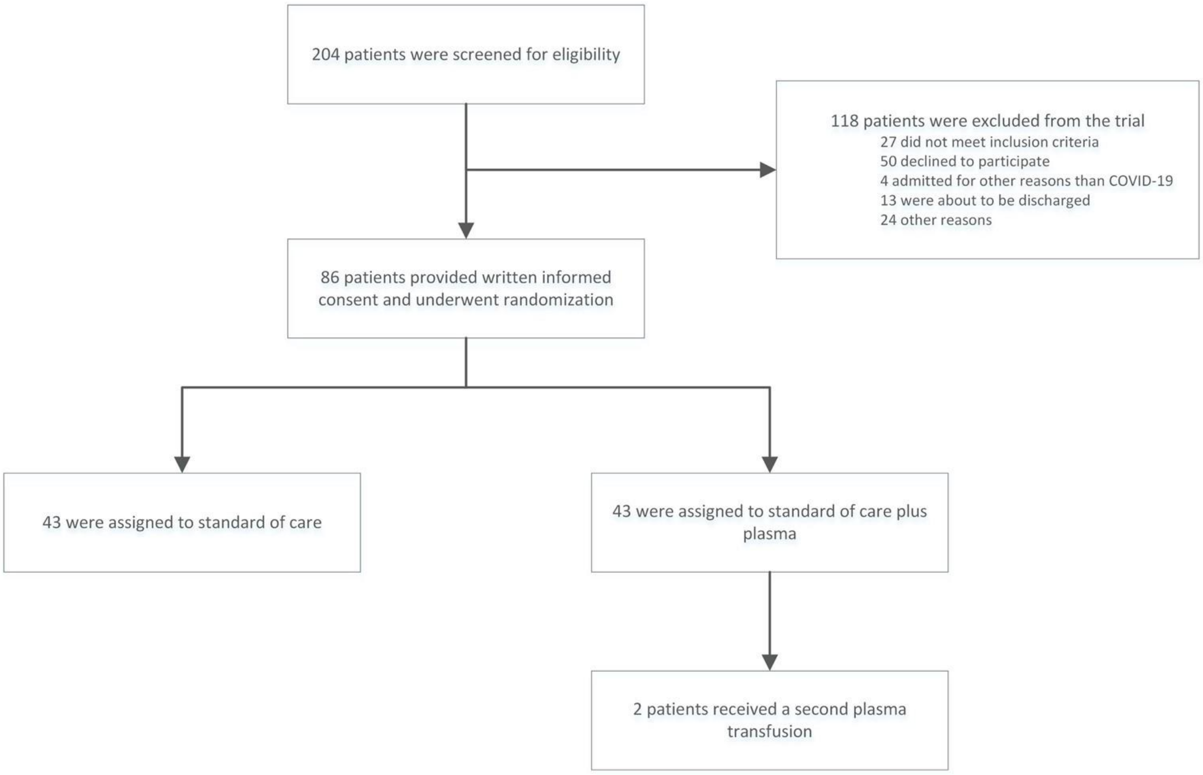

Supplementary Fig. 1. **Patient flowchart.**

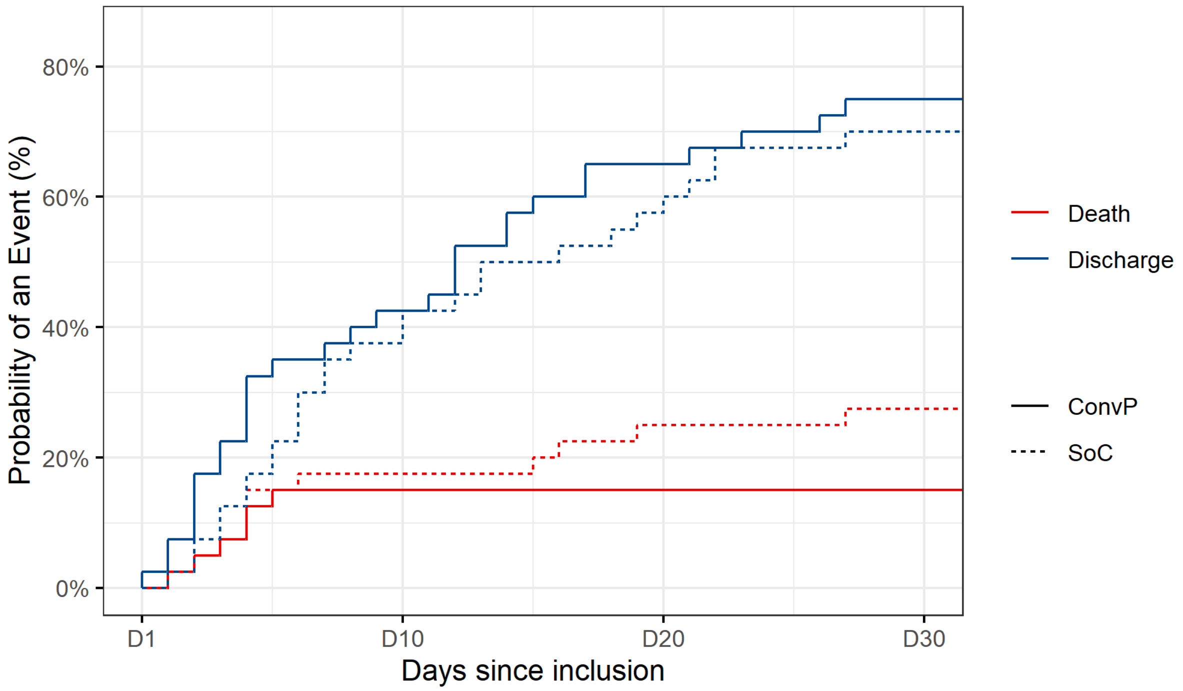

Supplementary Fig. 2. **Probability of discharge or death in patients.** Cumulative incidence curves of the sub-distribution of the probability of discharge (blue line) and death (red line) as competing risks for the two treatment groups (standard of care: dotted line, convalescent plasma: solid line) after enrollment (D=1).

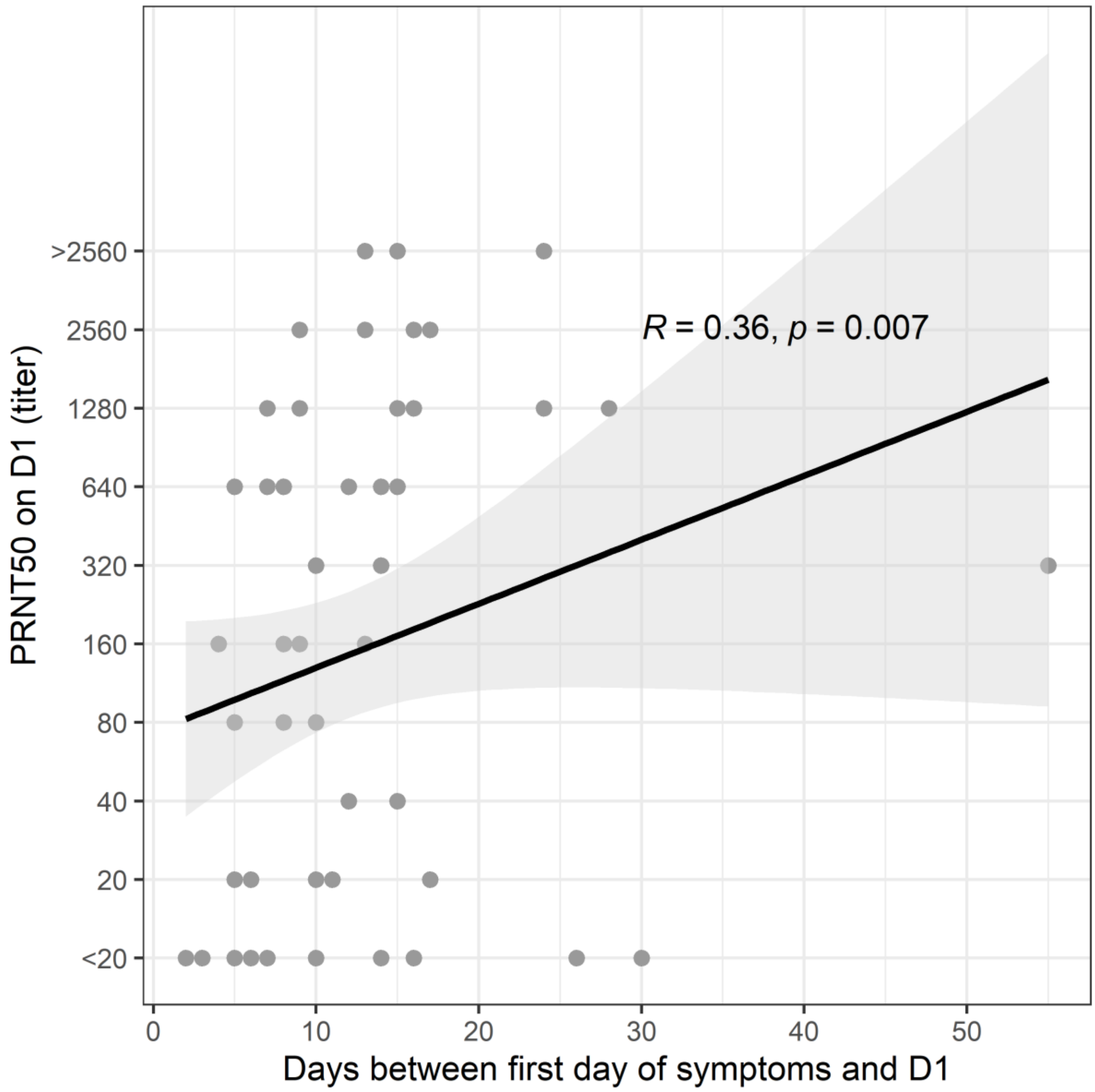

Supplementary Fig. 3. **Correlation between PRNT50 titer and symptoms.**

Spearman's rho with 2-tailed significance was used to evaluate the correlation (solid line) and 95% CI (shaded area) between PRNT50 titer measured on day 1 of enrollment and days of symptoms at time of enrollment in COVID-19 patients with available PRNT50 titers (N=56).

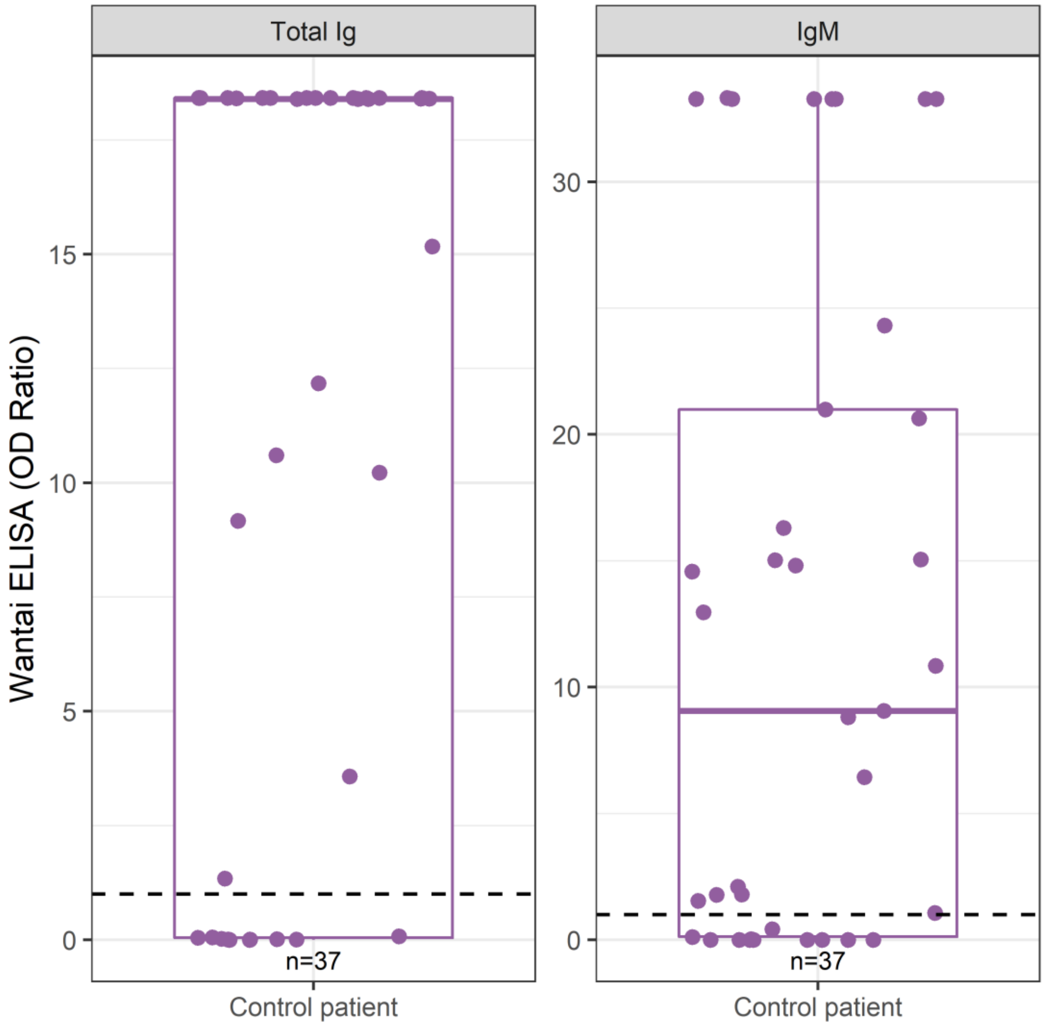

Supplementary Fig. 4. **ELISA OD ratio of total IG and IgM in control patients.**

SARS-CoV-2 total Ig and IgM against the receptor binding domain (RBD) measured by Wantai ELISA were evaluated in the serum of COVID-19 patients admitted for COVID19 in the weeks preceding the start of the study (N=37) and from whom serum was available. Serum was collected on the day of admission to the hospital.

Box plots indicate median (middle line), 25th, 75th percentile (box) and 5th and 95th percentile (whiskers) as well as outliers (single points). Dashed line indicates the positive cutt-off at 1.0 optical density (OD) ratio.

Supplementary Tables

Supplementary Table 1. **Baseline characteristics of COVID-19 patients of whom serum was analyzed for ELISA and PRNT50 titer.**

Analysed for:	Wantai Ig and IgM ELISA			PRNT50 titer
	SoC (n=32)	ConvP (n=34)	Total (n=66)	Total (n=56)
Male sex, n (%)	23 (72)	22 (65)	45 (68)	40 (71)
Age (years), median (IQR)	63 (55 – 73)	61 (56 – 69)	63 (55 – 72)	61 (54 – 68)
Duration of symptoms at inclusion (days), median (IQR)	12 (6 – 17)	9 (7 – 14)	10 (6 – 15)	10 (7 – 15)
Number of comorbidities, n (%)				
Diabetes Mellitus	6 (19)	11 (32)	17 (26)	11 (20)
Hypertension	9 (28)	9 (27)	18 (27)	13 (23)
Cardiac	7 (22)	7 (21)	14 (21)	11 (20)
Pulmonary	9 (28)	12 (35)	21 (32)	18 (32)
Cancer	1 (3)	4 (12)	5 (8)	4 (7)
Immunodeficiency	5 (16)	3 (9)	8 (12)	6 (11)
Chronic kidney disease	5 (16)	1 (3)	6 (9)	5 (9)
Liver cirrhosis	0	1 (3)	1 (2)	0
CRP (mg/L), median (IQR)	113 (73 – 209)	86 (50 – 134)	100 (51 – 163)	96 (49 – 134)
Ferritin (µg/L), median (IQR)	825 (644 – 1722)	522 (355 – 1051)	702 (445 – 1063)	721 (459 – 1062)
LDH (U/L), median (IQR)	386 (294 – 525)	336 (252 – 439)	366 (288 – 478)	374 (273 – 497)
Lymphocytes ($x10^9$/L), median (IQR)	0.95 (0.80 – 1.30)	1.10 (0.80 – 1.50)	1.00 (0.80 – 1.43)	1.00 (0.80 – 1.50)
Bilirubin (µmol/L), median (IQR)	9 (6 – 14)	8 (5 – 12)	8 (6 – 13)	9 (6 – 14)
WHO COVID-19 disease severity score[1], n (%)				
≤2	0	0	0	0
3	0	6 (18)	6 (9)	5 (9)
4-5	25 (78)	24 (81)	49 (74)	42 (75)
6-7	7 (22)	4 (12)	11 (17)	9 (16)

[1] WHO COVID-19 8-point Disease Severity Scale (at study inclusion for patients and highest score ever during disease course for donors): **0** = No clinical or virological evidence of infection, **1** = No limitation of activities, **2** = Limitation of activities, **3** = Hospitalized, no oxygen therapy, **4** = Oxygen by mask or nasal prongs, **5** = Non-invasive ventilation or high-flow oxygen, **6** = Intubation and mechanical ventilation, **7** = Ventilation + additional organ support = vasopressors, renal replacing therapy, extracorporeal membrane oxygenation (ECMO), **8** = Death.

Supplementary Table 2. **Odds ratios and 95% CIs from unadjusted and adjusted analysis of mortality.**

	Unadjusted OR (95% CI)	Adjusted OR (95% CI)	P-value
ConvP	0.472 (0.148; 1.384)	0.948 (0.195; 4.670)	0.95*
Age	-	1.097 (1.034; 1.179)	0.005
Female sex	-	0.717 (0.118; 3.932)	0.70
ICU on admission	-	6.701 (0.209; 142.602)	0.22
CRP on admission	-	1.000 (0.991; 1.009)	0.93
Lymphocyte count on admission	-	0.114 (0.013; 0.609)	0.023
Fi02 on admission	-	1.022 (0.994; 1.054)	0.13
Bilirubine	-	0.873 (0.727; 0.986)	0.078

*P-value from univariate Wald tests based on multivariable adjusted logistic regression

Supplementary Table 3. **Unadjusted and Adjusted Logistic Regression for 8-point WHO COVID-19 disease severity scale changes at day 15.**

	Unadjusted OR (95% CI)	Adjusted OR (95% CI)	P-value
ConvP	0.585 (0.273; 1.239)	1.300 (0.519; 3.318)	0.58*
Age	-	1.054 (1.017; 1.095)	0.006
Female sex	-	0.991 (0.345; 2.834)	0.99
ICU on admission	-	8.990 (1.566; 59.097)	0.017
CRP on admission	-	1.004 (0.998; 1.010)	0.16
Lymphocyte count on admission	-	0.189 (0.060; 0.546)	0.003
Fi02 on admission	-	1.030 (1.012; 1.050)	0.001
Bilirubin	-	0.982 (0.930; 1.036)	0.50

*P-value from univariate Wald tests based on multivariable adjusted logistic regression

Supplementary Table 4. **Unadjusted and Adjusted Logistic Regression for 8-point WHO COVID-19 disease severity scale changes at day 30.**

	Unadjusted OR (95% CI)	Adjusted OR (95% CI)	P-value
ConvP	0.555 (0.256; 1.189)	1.211 (0.497; 2.988)	0.67*
Age	-	1.061 (1.024; 1.103)	0.002
Female sex	-	0.594 (0.203; 1.728)	0.34
ICU on admission	-	2.473 (0.283; 21.28)	0.41
CRP on admission	-	1.002 (0.996; 1.009)	0.45
Lymphocyte count on admission	-	0.152 (0.046; 0.457)	0.001
Fi02 on admission	-	1.023 (1.004; 1.043)	0.019
Bilirubin	-	0.946 (0.885; 1.009)	0.098

*P-value from univariate Wald tests based on multivariable adjusted logistic regression

Supplementary Table 5. **Unadjusted Fine & Gray Regression for Time-to-Discharge**

	Unadjusted HR (95% CI)	Adjusted HR (95% CI)	P-value
ConvP	1.332 (0.811; 2.187)	0.883 (0.487; 1.603)	0.68*
Age	-	0.965 (0.947; 0.985)	<.001[%]
Female sex	-	1.166 (0.593; 2.290)	0.66
ICU on admission	-	0.218 (0.069; 0.694)	0.010
CRP on admission	-	0.998 (0.994; 1.002)	0.27
Lymphocyte count on admission	-	3.028 (1.495; 6.135)	0.002
Fi02 on admission	-	0.980 (0.968; 0.992)	0.002
Bilirubin	-	1.025 (0.991; 1.061)	0.15

*P-value from univariate Wald tests based on multivariable adjusted Fine and Gray regression

[%] Exact p-value: 0.0004933673

Supplementary Table 6. **Baseline characteristics of COVID-19 patients based on survival.**

	Total Alive (n=69)	Total Death (n=17)	SoC Death (n=11)	ConvP Death (n=6)
Male sex, n (%)	50 (73)	12 (71)	9 (82)	3 (50)
Age (years), median (IQR)	61 (53 – 69)	69 (63 – 84)	69 (63 – 81)	74 (64 – 87)
Duration of symptoms at inclusion (days), median (IQR)	10 (7 – 14)	6 (4 – 24)	6 (4 - 22)	6 (3 – 25)
Number of comorbidities, n (%)				
Diabetes Mellitus	15 (22)	6 (35)	2 (18)	4 (67)
Hypertension	16 (23)	6 (35)	4 (36)	4 67)
Cardiac	13 (19)	7 (41)	4 (36)	3 (50)
Pulmonary	20 (29)	3 (18)	3 (27)	0
Cancer	6 (9)	2 (12)	1 (9)	1 (17)
Immunodeficiency	10 (15)	1 (6)	0	1 (17)
Chronic kidney disease	5 (7)	2 (12)	2 (18)	0
Liver cirrhosis	1 (1)	0	0	0
CRP (mg/L), median (IQR)	97 (50 – 148)	126 (59 – 196	135 (73 – 241)	85 (48 – 159)
Ferritin (μg/L), median (IQR)	712 (425 – 1102)	532 (485 – 1026)	532 (487 – 1228)	622 (475 – 829)
LDH (U/L), median (IQR)	331 (266 – 454)	369 (299 – 534)	362 (293 – 540)	382 (296 – 503)
Lymphocytes (x10^9/L), median (IQR)	1.00 (0.88 – 1.50)	0.80 (0.55 – 1.15)	0.90 (0.50 – 1.00)	1.20 (0.63 – 1.40)
Bilirubin (μmol/L), median (IQR)	9 (6 – 14)	8 (5 – 10)	8 (5 – 11)	6 (5 – 9)
WHO COVID-19 disease severity score[(1)]				
≤2	0	0	0	0
3	8 (12%)	0	0	0
4-5	50 (72%)	15 (88%)	9 (82%)	6 (100%)
6-7	11 (16%)	2 (12%)	2 (18%)	0

[(1)] WHO COVID-19 8-point Disease Severity Scale (at study inclusion for patients and highest score ever during disease course for donors): **0** = No clinical or virological evidence of infection, **1** = No limitation of activities, **2** = Limitation of activities, **3** = Hospitalized, no oxygen therapy, **4** = Oxygen by mask or nasal prongs, **5** = Non-invasive ventilation or high-flow oxygen, **6** = Intubation and mechanical ventilation, **7** = Ventilation + additional organ support = vasopressors, renal replacing therapy, extracorporeal membrane oxygenation (ECMO), **8** = Death.

Supplementary Table 7. **Laboratory results in patients.**

The median CRP, ferritin and lymphocyte count at enrollment (day 1) and the median of the highest values of all CRP, ferritin and the median of the lowest values of all absolute lymphocyte counts between day 2-7 and day 8-14 after enrollment in both treatment groups. Data on CRP and ferritin were available in all 43 patients, data on absolute lymphocyte counts were available in 34 patients.

	SoC (n=43)	ConvP (n=43)	p-value
CRP day 1, median (IQR)	109 (70 – 165)	84 (50 – 133)	0.04
CRP day 2-7, median (IQR)	117 (50 – 261)	92 (44 – 237)	0.64
CRP day 8-14, median (IQR)	100 (34 – 156)	33 (17 – 146)	0.33
Ferritin day 1, median (IQR)	709 (525 – 1311)	702 (406 – 1060)	0.32
Ferritin day 2-7, median (IQR)	989 (525 – 2383)	1054 (737 – 1962)	0.68
Ferritin day 8-14, median (IQR)	716 (334 – 1686)	672 (466 – 1595)	0.75
Lymphocytes day 1 , median (IQR)	0.90 (0.74 – 1.30)	1.28 (1.00 – 1.67)	0.015
Lymphocytes day 2-7, median (IQR)	1.05 (0.70 – 1.68)	1.30 (9.2 – 1.57)	0.57
Lymphocytes day 8-14, median (IQR)	1.40 (0.82 – 2.24)	1.80 (1.55 – 2.50)	0.28

P-value from two-sided Mann-Whitney U-test

Supplementary Table 8. **Baseline characteristics of donors.**

	Donors[2] (n=115)	Donors selected for ConvP (n=19)
Male sex, n (%)	105 (91)	19 (100)
Age (years), median (IQR)	43 (31 – 52)	49 (38 – 54)
Duration after symptom resolution (days), median (IQR)	34 (22 – 42)	20 (15 – 25)
Number of comorbidities, n (%)		
Diabetes Mellitus	1 (1)	0
Hypertension	5 (5)	1 (6)
Cardiac	1 (1)	0
Pulmonary	6 (6)	0
Cancer	0	0
Immunodeficiency	1 (1)	0
Chronic kidney disease	0	0
Liver cirrhosis	0	0
WHO COVID-19 disease severity score[1]		
≤2	88 (88)	19 (100)
3	2 (2)	0
4-5	10 (10)	0
6-7	0	0

[1] WHO COVID-19 8-point Disease Severity Scale (at study inclusion for patients and highest score ever during disease course for donors): **0** = No clinical or virological evidence of infection, **1** = No limitation of activities, **2** = Limitation of activities, **3** = Hospitalized, no oxygen therapy, **4** = Oxygen by mask or nasal prongs, **5** = Non-invasive ventilation or high-flow oxygen, **6** = Intubation and mechanical ventilation, **7** = Ventilation + additional organ support = vasopressors, renal replacing therapy, extracorporeal membrane oxygenation (ECMO), **8** = Death.

Supplementary Table 9. **Baseline characteristics of control patients.**

	Total (n=37)
Male sex, n (%)	22 (60)
Age (years), median (IQR)	65 (55 – 74)
Duration of symptoms at inclusion (days), median (IQR)	10 (4 – 13)
Number of comorbidities, n (%)	
Diabetes Mellitus	7 (19)
Hypertension	14 (38)
Cardiac	12 (32)
Pulmonary	10 (27)
Cancer	6 (16)
Immunodeficiency	5 (14)
Chronic kidney disease	2 (5)
Liver cirrhosis	0
CRP (mg/L), median (IQR)	127 (38 – 241)
Ferritin (µg/L), median (IQR)	673 (467 – 870)
LDH (U/L), median (IQR)	331 (269 – 457)
Lymphocytes ($x10^9$/L), median (IQR)	0.91 (0.62 – 1.40)
Bilirubin (µmol/L), median (IQR)	8 (6 – 11)
WHO COVID-19 disease severity score[(1)]	
≤2	1 (3)
3	6 (16)
4-5	27 (73)
6-7	3 (8)

[(1)] WHO COVID-19 8-point Disease Severity Scale (at study inclusion for patients and highest score ever during disease course for donors): **0** = No clinical or virological evidence of infection, **1** = No limitation of activities, **2** = Limitation of activities, **3** = Hospitalized, no oxygen therapy, **4** = Oxygen by mask or nasal prongs, **5** = Non-invasive ventilation or high-flow oxygen, **6** = Intubation and mechanical ventilation, **7** = Ventilation + additional organ support = vasopressors, renal replacing therapy, extracorporeal membrane oxygenation (ECMO), **8** = Death.

Supplementary Table 10. **Cytokine concentrations in patients and healthy controls.**

Repeated measures of IL-1 β, IL-2, IL4, IL-10 and IL-12p70 in both treatments (SoC: N=10, ConvP: N=9) and in healthy controls.

	Day 1		Day 7		Day 14		Healthy controls
	SoC	ConvP	SoC	ConvP	SoC	ConvP	
IL-1β (pg/ml), mean (SD)	0.50 (± 0.08*)	0.46 (± 0.08)	0.56 (± 0.12)	0.42 (± 0.10)	0.60 (± 0.25)	0.49 (± 0.15)	ND
IL-2 (pg/ml), mean (SD)	0.94 (± 0.17)	0.73 (± 0.09)	0.70 (± 0.06)	0.81 (± 0.17)	ND	0.78 (± 0.14)	ND
IL-4 (pg/ml), mean (SD)	ND^	ND	ND	ND	ND	ND	ND
IL-10 (pg/ml), mean (SD)	21.43 (± 6.20)	25.49 (± 13.49)	9.95 (± 1.46)	14.50 (± 5.72)	6.91 (± 1.47)	12.97 (± 6.03)	2.21 (± 0.23)
IL-12p70 (pg/ml), mean (SD)	ND	ND	ND	0.62 (± 0.16)	ND	ND	ND

^ ND: not detected (below lower limit of quantitation)

Supplementary Table 11. **Mixed model for SARS-CoV-2 viral load in patients.** Estimates (95% CI) and p-values from univariate Wald tests based on the multivariable mixed-effects model for log(viral load) in log(copies/ml). The number of copies per ml was log-transformed in order to avoid deviations from normality assumptions. The value of 0.001 was added to zero values of viral load before the transformation to avoid minus infinity values after the log transformation.

	Estimate	P-value
(Intercept)	9.699 (0.379; 19.02)	0.042
Time	-0.811 (-1.107; -0.516)	<0.01[%]
ConvP	-5.507 (-9.920; -1.093)	0.016
Age	-0.058 (-0.177; 0.061)	0.33
Female sex	0.457 (-2.97; 3.885)	0.79
ICU on admission	0.619 (-4.453; 5.691)	0.81
CRP on admission	-0.016 (-0.037; 0.006)	0.15
Lymphocyte count on admission	-0.19 (-3.69; 3.31)	0.91
Fi02 on admission	0.039 (-0.017; 0.096)	0.17
Bilirubin	-0.095 (-0.261; 0.072)	0.26
Interaction between Time and ConvP	0.407 (0.011; 0.803)	0.044

[%] Exact p-value: 0.0000003652
