# Supplementary material for: Effects of potent neutralizing antibodies from convalescent plasma in patients hospitalized for severe SARS-CoV-2 infection
Source: Nat Commun. 2021 May 27;12:3189. doi: 10.1038/s41467-021-23469-2 (PMC8160346; doi:10.1038/s41467-021-23469-2)
Supplement: Supplementary file 4 — Reporting Summary [file 41467_2021_23469_MOESM4_ESM.pdf]

nature research

Corresponding author(s): Casper Rokx

Last updated by author(s): Feb 25, 2021

Reporting Summary

Nature Research wishes to improve the reproducibility of the work that we publish. This form provides structure for consistency and transparency in reporting. For further information on Nature Research policies, see our Editorial Policies and the Editorial Policy Checklist.

Statistics

For all statistical analyses, confirm that the following items are present in the figure legend, table legend, main text, or Methods section.

n/a	Confirmed	
☐	☒	The exact sample size (*n*) for each experimental group/condition, given as a discrete number and unit of measurement
☐	☒	A statement on whether measurements were taken from distinct samples or whether the same sample was measured repeatedly
☐	☒	The statistical test(s) used AND whether they are one- or two-sided<br>*Only common tests should be described solely by name; describe more complex techniques in the Methods section.*
☐	☒	A description of all covariates tested
☐	☒	A description of any assumptions or corrections, such as tests of normality and adjustment for multiple comparisons
☐	☒	A full description of the statistical parameters including central tendency (e.g. means) or other basic estimates (e.g. regression coefficient) AND variation (e.g. standard deviation) or associated estimates of uncertainty (e.g. confidence intervals)
☒	☐	For null hypothesis testing, the test statistic (e.g. *F*, *t*, *r*) with confidence intervals, effect sizes, degrees of freedom and *P* value noted<br>*Give P values as exact values whenever suitable.*
☒	☐	For Bayesian analysis, information on the choice of priors and Markov chain Monte Carlo settings
☒	☐	For hierarchical and complex designs, identification of the appropriate level for tests and full reporting of outcomes
☐	☒	Estimates of effect sizes (e.g. Cohen's *d*, Pearson's *r*), indicating how they were calculated

Our web collection on statistics for biologists contains articles on many of the points above.

Software and code

Policy information about availability of computer code

Data collection	Data was collected in an eCRF using OpenClinica
Data analysis	Simple statistical tests were performed using SPSS (eg. chi-squared test). Graphs and complex statistics were performed using R-studio.

For manuscripts utilizing custom algorithms or software that are central to the research but not yet described in published literature, software must be made available to editors and reviewers. We strongly encourage code deposition in a community repository (e.g. GitHub). See the Nature Research guidelines for submitting code & software for further information.

Data

Policy information about availability of data

All manuscripts must include a data availability statement. This statement should provide the following information, where applicable:

- Accession codes, unique identifiers, or web links for publicly available datasets
- A list of figures that have associated raw data
- A description of any restrictions on data availability

We are willing to share all raw but anonymized data if allowed under the European GDRP regulations n reasonable request for non-profit organisations unless the data are already available in the manuscript.

Field-specific reporting

Life sciences study design

All studies must disclose on these points even when the disclosure is negative.

Sample size	86 subjects.
Data exclusions	1 subject at day 30 of follow-up refused further follow-up. Other than that all follow-up was performed according to protocol
Replication	*Describe the measures taken to verify the reproducibility of the experimental findings. If all attempts at replication were successful, confirm this OR if there are any findings that were not replicated or cannot be reproduced, note this and describe why.*
Randomization	Subjects were randomized without stratification using an online randomization system using blocks of 4 and 2 in ALEA
Blinding	Subjects were allocated to standard of care with or without convalescent plasma. Blinding was not possible in this setup.

Reporting for specific materials, systems and methods

We require information from authors about some types of materials, experimental systems and methods used in many studies. Here, indicate whether each material, system or method listed is relevant to your study. If you are not sure if a list item applies to your research, read the appropriate section before selecting a response.

Materials & experimental systems

n/a	Involved in the study	
☐	☒	Antibodies
☒	☐	Eukaryotic cell lines
☒	☐	Palaeontology and archaeology
☒	☐	Animals and other organisms
☐	☒	Human research participants
☐	☒	Clinical data
☒	☐	Dual use research of concern

Methods

n/a	Involved in the study	
☒	☐	ChIP-seq
☒	☐	Flow cytometry
☒	☐	MRI-based neuroimaging

Antibodies

Antibodies used	Anti-SARS-CoV-2 antibodies in units of plasma retrieved from donors using plasmaphereris
Validation	All units of plasma were tested for anti-SARS-CoV-2 antibodies using a Wantai ELISA and PRNT50.

Human research participants

Policy information about studies involving human research participants

Population characteristics	Hospitalized patients with PCR confirmed COVID-19, aged 18 or older, with the most recent PCR < 96 hours old.<br>No known IgA defieciency. No participation in another COVID-19 trial. No invasive ventilation for already > 96 hours.
Recruitment	Eligible patients in hospital or their legal representatives were approached in hospital for an informed consent conversation by a member of the study team.
Ethics oversight	Ethical commission of Erasmus MC Rotterdam

Note that full information on the approval of the study protocol must also be provided in the manuscript.

Clinical data

Policy information about clinical studies

All manuscripts should comply with the ICMJEguidelines for publication of clinical research and a completedCONSORT checklist must be included with all submissions.

Clinical trial registration	Clinicaltrials.gov: NCT04342182
Study protocol	https://www.medrxiv.org/content/10.1101/2020.07.01.20139857v1
Data collection	From the 8th of April to the 14th of June 2020, subjects were enrolled from 12 dutch hospitals
Outcomes	Primary outcome: overall mortality until discharge or a maximum of 60 days using a multivariable logistic regression model.
